# Supplementary material for: Molecular Diagnosis of Koala Retrovirus (KoRV) in South Australian Koalas (Phascolarctos cinereus)
Source: Animals (Basel). 2021 May 20;11(5):1477. doi: 10.3390/ani11051477 (PMC8161083; doi:10.3390/ani11051477)
Supplement: Supplementary file 1 [file animals-11-01477-s001.zip › animals-1195664-supplementary.pdf]

animals

MDPI

Supplementary Materials

Molecular Diagnosis of Koala Retrovirus (KoRV) in South Australian Koalas (*Phascolarctos cinereus*)

Tamsyn Stephenson [1,*], Natasha Speight [1], Wai Yee Low [2], Lucy Woolford [1,3], Rick Tearle [2] and Farhid Hemmatzadeh [1]

Table S1. Comparative CT method results for RNA from lymph nodes of koalas that were KoRV positive and KoRV positive diagnosed with lymphoma, using KoRV positive as the control group.

Sample and Gene	KoRV Gene Average C_T	ACT-G Average C_T	ΔC_T KoRV Gene-ACT-G	$\Delta\Delta C_T$ ΔC_T KoRV Group -ΔC_T KoRV Positive (Control)	$2^{-\Delta\Delta CT}$ Fold difference in KoRV Gene Relative to KoRV Negative * Mean (Range)	Z Score	*p* val
Positive *gag* 1	22.14	20.57	1.57	0.00	1 (0.1–7.0)		
Lymphoma *gag* 1	20.01	20.88	−0.87	−2.44	5.4 (0.9–32.2)	−1.46	0.144
Positive *gag* 2	27.27	20.57	6.70	0.00	1 (0.0–27.6)		
Lymphoma *gag 2*	19.36	20.88	−1.52	−8.23	299 (47.7–1880.5)	−1.88	0.060
Positive *pol*	30.41	20.57	9.84	0.00	1 (0.1–15.2)		
Lymphoma *pol*	21.49	20.88	0.61	−9.23	602 (185.8–1950.2)	−2.51	0.012
Positive *env* 1	26.04	20.57	5.47	0.00	1 (0.1–13.2)		
Lymphoma *env* 1	18.44	20.88	−2.45	−7.92	242 (42.5–1375.6)	−2.51	0.012

* bold values are significant (Mann-Whitney U test, $p < 0.05$).

Table S2. BLASTn analysis results of KoRV termini regions to the koala reference genome. KoRV is represented by the reference genome AF151794. with alignment to the koala reference genome (phaCin_unsw_v4.1 Annotation Release 100; GCF_002099425.1) Adapted from hit table produced from NCBI (blast.ncbi.nlm.nih.gov).

Scaffold	Start	Stop	Length	Aligned	% Ident
scaf00137	4,943,695	4,944,883	1189	1178	99.07%
	4,938,042	4,938,853	812	811	99.88%
scaf00005	26,678,572	26,679,758	1187	1176	99.07%
	26,672,908	26,673,719	812	811	99.88%
scaf00288	726,500	727,680	1181	1172	99.24%
	732,519	733,330	812	811	99.88%
scaf00113	3,973,843	3,975,031	1189	1177	98.99%
	3,968,195	3,969,005	814	806	99.02%
scaf00073	4,193,643	4,194,823	1181	1171	99.15%
	4,199,659	4,200,470	812	811	99.88%
scaf00357	128,022	129,200	1181	1170	99.07%
	134,041	134,852	812	811	99.88%
scaf00253	2,925,255	2,926,428	1181	1165	98.65%
	2,931,284	2,932,088	805	804	99.88%

Table S3. BLASTn analysis results of PhER to the koala reference genome: PhER: phaCin_unsw_v4.1. fa.scaf00062:10912078-10920108, as per Lober et al. 2018 [20] and the reference koala genome: phaCin_unsw_v4.1 Annotation Release 100; GCF_002099425.1. Adapted from hit table produced from NCBI (blast.ncbi.nlm.nih.gov).

Scaffold	Start	Stop	Length	Aligned	% Ident
scaf00137	4,939,133	4,943,711	4588	4414	96.21%
	4,945,223	4,945,551	329	314	95.44%
scaf00005	26,673,999	26,678,588	4594	4415	96.10%
	26,673,752	26,674,080	329	314	95.44%
scaf00288	727,670	732,238	4585	4411	96.21%
	732,157	732,486	330	315	95.45%
scaf00113	3,969,286	3,973,859	4586	4420	96.38%
	3,969,038	3,969,367	330	315	95.45%
scaf00073	4,194,813	4,199,379	4580	4417	96.44%
	4,199,298	4,199,626	332	315	94.88%
scaf00357	129,190	133,760	4583	4415	96.33%
	133,679	134,008	330	315	95.45%
scaf00253	2,926,418	2,931,005	4590	4418	96.25%
	2,930,924	2,931,251	329	313	95.14%
